# Sexual Absorption of Vaginal Progesterone: A Randomized Control Trial

**DOI:** 10.1155/2015/685281

**Published:** 2015-02-03

**Authors:** Kathryn S. Merriam, Kristina A. Leake, Mollie Elliot, Michelle L. Matthews, Rebecca S. Usadi, Bradley S. Hurst

**Affiliations:** Division of Reproductive Endocrinology and Infertility, Department of Obstetrics and Gynecology, Carolinas Medical Center, 1000 Blyth Boulevard, Charlotte, NC 28203, USA

## Abstract

*Objective.* To determine if sexual intercourse reduces absorption of vaginal progesterone gel in women and to determine if progesterone is absorbed by the male during intercourse. *Study Design.* Prospective, randomized, cross over, controlled study of 20 reproductive-aged women and their male sexual partners randomized to receive vaginal progesterone gel (Crinone 8% gel, Actavis Inc., USA) or placebo cream. Serum progesterone for both male and female partners were measured 10 hours after intercourse. One week later, subjects were crossed over to receive the opposite formulation. In the third week, women used progesterone gel at night and abstained from intercourse. *Results.* Serum progesterone was significantly reduced with vaginal progesterone gel + intercourse compared with vaginal progesterone gel + abstinence (*P* = 0.0075). Men absorbed significant progesterone during intercourse with a female partner using vaginal progesterone gel compared to placebo (*P* = 0.0008). *Conclusion(s).* Vaginal progesterone gel is reduced in women after intercourse which may decrease drug efficacy during luteal phase support. Because men absorb low levels of progesterone during intercourse, exposure could cause adverse effects such as decreased libido. This study is registered under Clinical Trial number NCT01959464.

## 1. Introduction

Vaginal progesterone cream may be used by women for several clinical indications, and if intercourse alters the absorption and distribution of vaginal progesterone, clinical outcomes may be compromised. Progesterone supplementation is commonly used for luteal phase support in assisted reproductive technology (ART) as it has been shown to increase ongoing pregnancy rates [1]. ART outcomes have been shown to be equivalent when using vaginal progesterone gel and intramuscular progesterone, but vaginal progesterone tends to be better tolerated by women [2, 3]. Additionally, vaginal progesterone has been shown to reduce the rate of spontaneous premature delivery before 34 weeks in women with a short cervix [4].

Male absorption of progesterone could have adverse effects. Progesterone therapy was introduced in the 1960s as a potential treatment of men who were convicted as sexual offenders and paraphilias. Medroxyprogesterone use in men was reported to temporarily decrease serum testosterone and gonadotropin levels resulting in decreased libido, frequency of erection, and spermatogenesis [5–7]. Increased amounts of progesterone in men whose partners are using vaginal progesterone gel could have similar consequences.

The effects of intercourse on the absorption of vaginal progesterone for the female user and her sexual partner have not been studied. However, a previous study performed by our group found that intercourse lowered the absorption of vaginal estrogen cream in women, and men absorbed a small but statistically significant amount of estradiol during intercourse [8].

The purpose of this study is to determine if sexual intercourse lowers serum progesterone levels in women using vaginal progesterone gel and, secondly, to determine if sexual intercourse increases serum progesterone levels in these women's male sexual partners.

## 2. Materials and Methods

This was a prospective, randomized, placebo-controlled, blinded, crossover study of 20 reproductive-aged women and their male sexual partners, ages 18–40. Sample size was based on a previous study performed by our group assessing vaginal estrogen absorption; the sample size was doubled from the previous study to achieve adequate power. A sample size of 20 achieved 84% power to detect a difference of 0.07 ng/mL between the null hypothesis of a median progesterone level of 0.135 ng/mL and the alternative hypothesis median of 0.065 ng/mL (after coitus) with an estimated standard deviation of 0.1 ng/mL.

After institutional review board approval was obtained (Carolinas HealthCare System IRB #11-08-03A) and study consent forms were signed, the couples were randomized to receive vaginal progesterone gel (Crinone 8% gel, Actavis Inc., USA) or placebo while the female participant was taking a monophasic oral contraceptive (Mircette, Teva Pharmaceuticals, USA—0.15 mg desogestrel and 0.02 mg ethinyl estradiol) to suppress ovulation [9, 10]. Mircette was used to minimize variability in progesterone levels during the study; a prior unpublished internal study demonstrated that the progestin desogestrel had no cross reactivity with the serum progesterone assay used.

**Figure 1:**
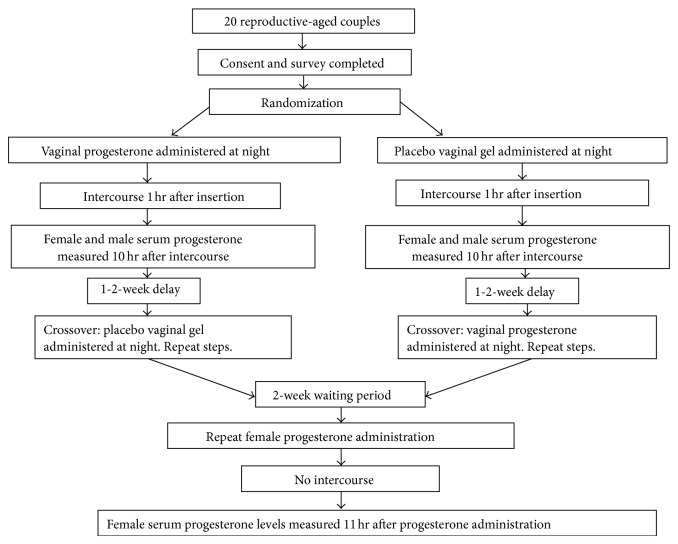
Study methods.

The study medication and placebo were assembled by the pharmacy, blinded to the investigators and women, and given to couples in computer-generated random order. The progesterone (or placebo) was administered at night, 1 hour before intercourse, and serum progesterone was measured for the subject and her partner 10 hours after intercourse. One week later, the subjects were crossed over to receive the opposite formulation, but the same protocol was used. In the third week, women used progesterone gel at night and abstained from intercourse, and blood was drawn 11 hours later. The time of insertion of the cream and the time of intercourse (when applicable) were recorded to the nearest half hour on a data sheet and returned to the study nurse via preaddressed and stamped envelopes (Figure 1). The full protocol can be found online at http://clinicaltrials.gov/show/NCT01959464.

Inclusion criteria included sexually active heterosexual women and their partners 18–40 years old, willing to take Mircette birth control pills for at least one cycle, willing to have intercourse and blood draws at defined times, and willing to sign informed consent. Exclusion criteria included contraindications for oral contraceptives, known sensitivity to vaginal progesterone gel, use of condoms during intercourse, or male erectile or ejaculatory dysfunction.

Recruitment of study participants occurred between November 2008 and September 2010. The trial ended when all 20 couples completed the entire three-week protocol of the study.

Descriptive statistics including mean, medians, standard deviations, and interquartile ranges were calculated. Shapiro-Wilk tests were used to test for normality. Several variables were not normally distributed; therefore, nonparametric tests were used. The Friedman two-way analysis of variance by ranks was used to determine if the distributions of the serum progesterone levels are different for the three treatment groups. Wilcoxon matched-pairs signed rank tests were used to compare the serum progesterone levels for the different treatment groups. The Wilcoxon rank sum test compared serum progesterone levels between women who received vaginal progesterone gel in the first time period and women who received vaginal progesterone gel in the second time period. Similarly, we also compared women who received the placebo gel in the first time period with women who received the placebo gel in the second time period. The order of treatments was not found to affect serum progesterone measurements for the two groups. SAS Enterprise Guide, version 5.1, was used for all analyses. A two-tailed *P* value of less than 0.05 was considered statistically significant.

## 3. Results

**Figure 2:**
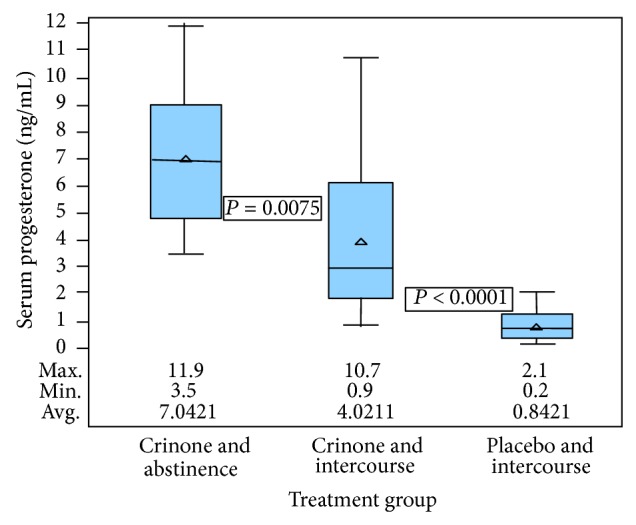
Distribution of female serum progesterone by treatment group. Crinone and abstinence group: maximum 11.9, minimum 3.5, and average 7.042. Crinone and intercourse group: maximum 10.7, minimum 0.9, and average 4.021. Placebo and intercourse group: maximum 2.1, minimum 0.2, and average 0.842.

When vaginal progesterone gel was used, intercourse markedly reduced serum progesterone levels (2.9 ng/mL [1.9–6.2]) compared to abstinence (6.9 ng/mL [4.8–9.0]; *P* = 0.0075) (Table 1 and Figure 2). As expected, serum progesterone levels were significantly higher with vaginal progesterone administration compared to the vaginal placebo (median 6.9 ng/mL and interquartile range [4.8–9.0] compared with 0.8 [0.3–1.3]; *P* < 0.0001) (Figure 2). Serum progesterone with placebo and intercourse was significantly less than vaginal progesterone gel and intercourse (median 0.8 ng/mL and interquartile range [0.3–1.3] compared with 2.9 ng/mL [1.9–6.2]; *P* < 0.0001).

**Figure 3:**
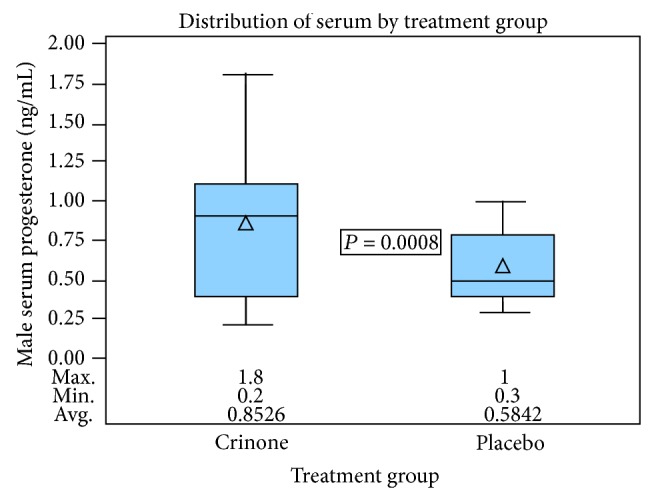
Distribution of male serum progesterone by treatment group. Crinone group: maximum 1.8, minimum 0.2, and average 0.853. Placebo group: maximum 1, minimum 0.3, and average 0.584.

Serum progesterone levels were significantly higher in men whose female partners used vaginal progesterone gel compared with men whose female partners used placebo (0.9 ng/mL [0.4–1.1] compared to 0.5 ng/mL [0.4–0.8]; *P* = 0.0008) (Figure 3).

The final sample size was 19 couples; 1 couple moved out of town prior to completing the study. Demographic information is provided in Table 1. Each analysis included all 19 couples because it was a crossover study. No harms or unintended effects occurred during the trial.

## 4. Discussion 

This study demonstrates that intercourse lowers serum progesterone significantly in women using vaginal progesterone gel. Progesterone levels were not different between 1st and 2nd cycles in the crossover design in either group.

Based on these results, it is possible that intercourse during luteal phase support could decrease drug efficacy in women using vaginal progesterone. It is well known that progesterone supports embryo implantation and helps to maintain early pregnancies [2, 9]. Progesterone supplementation during luteal phase after ART has been shown to increase ongoing pregnancy rates [1]. Vaginal progesterone has also been shown to be effective in reducing rates of spontaneous preterm deliveries in women with asymptomatic short cervix [4]. Therefore, a decrease in drug absorption with intercourse could lead to reduced pregnancy rates and increased risks of miscarriage in women with otherwise successful fertility treatment, as well as increased rates of preterm delivery in women with a short cervix.

The exact mechanism which causes decreased absorption of vaginal progesterone during intercourse is unclear, and it is outside the scope of this study. Possible explanations may include absorption by the male partner, dilution effect from vaginal secretions or seminal fluid, or a rapid spike followed by rapid decline in progesterone levels due to increased blood flow to the vagina during intercourse. Further studies regarding the pharmacokinetic changes over time after intercourse in both the male and female partners would be necessary to further elucidate the answer. Regardless, the observation that intercourse significantly lowers progesterone levels when vaginal progesterone cream is used is of concern, since intercourse may lower the efficacy of the drug.

Men absorb low but significant levels of progesterone after intercourse with a female partner using vaginal progesterone. Male exposure to progesterone during the typical 8–12 weeks used during early pregnancy could cause adverse effects such as decreased libido. When progesterone (medroxyprogesterone) was used for treatment of male sex offenders starting in the 1960s, men were noted to have reduced sexual thoughts and fantasies, frequency and pleasure of masturbation, and frequency of early morning erections on awakening [6]. Although vaginal progesterone gel is not similar to medroxyprogesterone, the effects of vaginal progesterone gel have not been directly studied in men and therefore are not known.

These results are consistent with results of a previous study performed by our group which showed that intercourse lowered the absorption of vaginal estrogen cream in women, and men absorbed a small but statistically significant amount of estradiol during intercourse [8].

Strengths of this study include being both blinded and a crossover study. Furthermore, our sample size was chosen based on power calculation and was sufficient to identify changes in progesterone levels. Limitations include use of serum progesterone as a measure of vaginal progesterone absorption, no examination of delayed intercourse, and not knowing the clinical significance of decreased serum progesterone in women who use vaginal progesterone.

We chose to use serum progesterone levels to measure the absorption of vaginal progesterone gel in women and their male sexual partners. Because of their wide range and fluctuations, serum progesterone levels have not been proven to have clinical utility in determining luteal phase deficiency [11, 12]. Another measure of progesterone absorption is histologic assessment of the endometrium, but endometrial histology has also proven to be invalid measure of luteal phase deficiency [11, 12]. There are alternative measurements of absorption such as measuring endometrial progesterone levels in endometrial biopsy samples or assessing binding of endometrial progesterone receptors [13, 14]. Serum progesterone measurements were chosen for this study because they are less painful and less expensive for the research volunteers than endometrial biopsy procedure. The aim of this study was to determine progesterone absorption as it is altered by intercourse, which is adequately measured by serum progesterone. Further, variability in progesterone level was minimized using Mircette oral contraceptive, which does not cross react with the serum progesterone assay. This study did not look at the effect of delayed intercourse on absorption of vaginal progesterone. In many women, vaginal progesterone is administered in the morning, and intercourse may take place later in the evening. For ease of timing in our participants' schedules and improved participant compliance, we chose to have application of the progesterone and subsequent intercourse in the evening so that blood draws could be timed the following morning; this would allow time for the maximum serum progesterone concentrations to be obtained (6.8 +/− 3.3 hr with a single dose) [9].

We chose to use Crinone in this study as it is FDA-approved for luteal support [9]. Other forms of vaginal progesterone have been described in the literature [15]. Further research would be required to determine if our results apply to the use of other formulations.

The absolute treatment levels for pregnancy maintenance are not known, nor is it known what level of progesterone may impair male libido. While the absolute lowest serum progesterone level to achieve a viable pregnancy is unknown, one case report described first trimester progesterone levels of 1.2 ng/mL for a pregnancy that progressed to 27 weeks [16]. Vaginal progesterone provides levels that are clinically proven to be above the “threshold” to maintain a pregnancy, but a drop below this threshold is expected to cause miscarriage.

The concept of “first pass uterine effect” that indicates that the vaginal application delivers higher levels of progesterone to the endometrium than intramuscular injections has become a dogmatic belief by some, but several important clinical observations argue against this theory. The most widely cited study describing the “first pass uterine effect” studied serum to endometrial tissue levels in postmenopausal women who were undergoing hysterectomy, and even though the endometrial tissue levels of progesterone were not significantly different between the intramuscular progesterone group and the vaginal progesterone gel group, the ratios of serum to endometrial progesterone ratios were significantly higher with vaginal progesterone [17]. The difference in ratios should not be surprising, since serum levels are relatively low when vaginal progesterone is used.

There are several arguments against the “first pass uterine effect” theory. First, there is no vascular portal circulation from the vagina to the uterus. Vaginal progesterone must be absorbed by the capillaries in the vagina and then transported via veins, heart, and arteries before it is circulated to the uterus and the endometrium. It is clear from our data that serum progesterone levels are relatively low, compared to typical mid-luteal levels. If endometrial progesterone exposure is low with vaginal progesterone, it could be predicted that more spotting would occur with the use of vaginal progesterone and less with the use of intramuscular progesterone, and more spotting or light bleeding with vaginal progesterone use is exactly what has been shown to occur when used for IVF [2]. While clinical studies have shown that vaginal progesterone is adequate to achieve ART pregnancies and live births and can achieve outcomes comparable to intramuscular progesterone [2], it is concerning that intercourse could further lower the endometrial exposure to progesterone when it is used vaginally and possibly lower live birth rates.

Although we do not yet know the clinical significance of decreased vaginal progesterone as a result of intercourse, important conclusions regarding progesterone absorption for both women and men may be drawn from this novel randomized controlled study. The significantly decreased absorption of progesterone in women with intercourse may lead to adverse pregnancy outcomes for those undergoing ART and women with short cervix using vaginal progesterone to prevent preterm labor. Increased absorption in men after intercourse with women using vaginal progesterone gel, while not directly known, may have adverse effects on libido. These observations are especially troubling since there are only two progesterone formulations approved by the FDA, and both require vaginal administration. However, further studies are needed to elucidate the effect of decreased vaginal progesterone on ART outcomes and on premature delivery rates in women with short cervix as well as on male response to increased progesterone.

## Figures and Tables

**Table 1:** Baseline characteristics of study participants.

	Mean (range)
Age (female)	29.8 (21–39)
Gravida	0.8 (0–6)
Para	0.5 (0–3)
BMI (female)	24 (18.8–39.7)
BMI (male)	26 (21.3–31.3)

	*N* (percentage)

Ethnicity	
Caucasian	15 (78.9%)
African American	2 (10.5%)
Hispanic	1 (5.3%)
Other	1 (5.3%)
